# Gallic acid lessens kidney injury induced by inorganic arsenic and zinc oxide nanoparticles in rats via controlling electrolyte balance, oxidative stress, and Nrf-2 and HSP-90 expression

**DOI:** 10.1186/s40360-025-01044-5

**Published:** 2025-11-29

**Authors:** Yasmina M. Abd-Elhakim, Mohamed M. M. Hashem, Khaled Abo-EL-Sooud, Mohamed R. Mousa, Bayan A. Hassan

**Affiliations:** 1 Department of Forensic Medicine and Toxicology, Faculty of Veterinary Medicine, Zagazig University, Zagazig, 44519 Egypt; 2 Department of Pharmacology, Faculty of Veterinary Medicine, Cairo University, Giza, 12211 Egypt; 3 Department of Pathology, Faculty of Veterinary Medicine, Cairo University, Giza, 12211 Egypt; 4 Pharmacology Department, Faculty of Pharmacy, Future University, Cairo, 11835 Egypt

**Keywords:** Arsenic, Kidney, Zinc oxide nanoparticles, Gallic acid, HSP90, Nrf-2

## Abstract

Inorganic arsenical compounds, such as arsenic trioxide (ATO), are toxic environmental contaminants that occur widely in soil, water, and biological systems. Besides, zinc oxide nanoparticles (ZNPs) have been recently incorporated in various industrial and medicinal applications. Thus, their co-existence in the environment could widely occur. This study examined the potential protective activity of gallic acid (GA, 20 mg/kg b. wt) against the harmful impacts of 60-day co-exposure to ATO (8 mg ATO/kg b. wt) and ZNPs (100 mg ZNPs/kg b. wt) on the kidneys of rats. The results indicated that ZNPs and/or ATO exposure resulted in increased serum levels of markers associated with renal damage, an imbalance in electrolytes (sodium, potassium, and calcium), diminished levels of antioxidant enzymes in the kidneys, and an increased malondialdehyde (MDA) concentration. Furthermore, ZNPs and/or ATO co-exposed rats demonstrated markedly increased levels of renal zinc (Zn) and arsenic (As), accompanied by pronounced histopathological alterations, including interstitial nephritis, renal tubular necrosis, and vascular wall thickening. Immunohistochemical analysis revealed that exposure to ZNPs and/or ATO reduced the expression of nuclear factor erythroid 2-related factor 2 (Nrf2) while increasing that of heat shock protein 90 (HSP90) in kidney tissues. Co-exposure to ZNPs and ATO produced more pronounced alterations, including increased serum uric acid and creatinine, decreased sodium levels, reduced renal GPx activity, increased MDA content, greater renal accumulation of As and Zn, and diminished Nrf2 expression, compared with individual exposures, suggesting additive toxic effects. However, GA notably reduced renal tissue damage, oxidative stress, and disturbances in renal function and electrolyte balance in rats co-exposed to ZNPs and ATO. Conclusively, the study found that exposure to ZNPs and ATO, especially when combined, was toxic to the kidneys, leading to impaired renal function through increased oxidative stress and disrupted electrolyte balance. However, GA effectively protected kidney health at the administered doses by counteracting these effects through its antioxidant properties and by modulating cellular defense mechanisms involving Nrf2 and HSP90.

## Introduction

Arsenic (As) is a bioaccumulative persistent contaminant extensively found in groundwater and soil [1]. It is widely utilized in industrial processes associated with the production of diverse products, including agricultural pesticides, glass, pigments, and wood preservatives [2]. Numerous countries have used As-based pesticides, resulting in substantial environmental pollution and arsenic poisoning [3, 4]. Non-occupational As exposure occurs mostly through the inhalation of air, consumption of food, and ingestion of water in daily life. The ingestion of drinking water with increased As levels significantly contributes to arsenic poisoning [5]. The groundwater contamination of As in Bangladesh represents the largest mass poisoning in history, affecting millions of people [6, 7]. It may also be ingested through food [8]. Arsenic trioxide (ATO) is the predominant inorganic variant and is recognized for its toxicity and lethality to living organisms [9]. The kidney is known to accumulate environmental As [10]. The kidney is highly vulnerable to As exposure, significantly influencing As accumulation or excretion [11]. Consequently, the renal toxicity of As is garnering increasing interest [12, 13]. ATO exposure may generate nephrotoxicity by disrupting the antioxidant defense system and generating oxidative damage [9].

Zinc oxide nanoparticles (ZNPs) are important metal oxide NPs, exhibiting notable characteristics like chemical and physical stability, high catalytic activity, potent antibacterial properties, and substantial ultraviolet and infrared absorption [14]. Additionally, ZNPs have demonstrated potential as an effective insecticide for the control of several types of insects [15–17]. Yet, recent studies have indicated the adverse toxic effects of ZNPs, including a reduction in cell viability, cellular damage, apoptosis, and alterations in the cytoskeleton [18, 19]. In vitro investigations indicated that the potential mechanism underlying ZNPs toxicity involves increased reactive oxygen species (ROS) production, subsequently resulting in ROS-induced oxidative stress [18, 20]. As urinary excretion of ZNPs is the main route to clear out Zn, the ZNPs renal hazards received high interest [21]. In the recent study of Kim, et al. [22], ZNPs induced autophagy-mediated cell death in normal human kidney cells.

In real-world environments, As compounds and metal oxide NPs frequently coexist in contaminated soils, water systems, and agricultural waste, such as sewage sludge or industrial effluents [23]. The wide range of applications of ZNPs in commercial products and the ubiquitous heavy metal contamination in the natural environment increase the chance of the co-existence of ZNPs with various heavy metals in the surroundings [24]. This co-occurrence suggests that simultaneous exposure to ATO and ZNPs is environmentally relevant and may pose compound health risks [25]. Moreover, co-exposure to ZNPs and ATO is of toxicological importance because nanomaterials like ZNPs can act as carriers or modulators of heavy metal ions, potentially enhancing their cellular uptake and retention [26–28]. These interactions suggest a possible synergistic or additive effect during co-exposure, particularly in renal tissues, which are primary sites for the clearance and accumulation of both Zn [29] and As [30].

In several toxicological studies, the effects of ZNPs have been investigated in combination with metals in their micro-sized forms [24, 31, 32]. Differences in the physicochemical properties between metal oxides in nanoform and heavy metals in microform may influence their biological behavior. For example, nano-ZNPs may disrupt lysosomes or mitochondria [33], while micro-ATO may act via solubilization and transport as arsenite ions [34]. Besides, NPs, due to their large surface area and high reactivity, can penetrate biological barriers more easily and may alter the toxicokinetics of co-present microcontaminants such as ATO [35]. Moreover, in this study, the choice to use micro-As and nano-Zn reflects their prevalent environmental or occupational forms [36, 37]. Therefore, exploring this combined exposure model can offer deeper insights into real-world risk assessments.

Increasing research has underlined the significance of using natural antioxidants to reduce oxidative renal damage resulting from environmental pollutants [38–40]. Gallic acid (GA, 3,4,5-trihydroxybenzoic acid), a naturally occurring phenolic compound, has gained attention for its powerful antioxidant [41]. Notably, GA can chelate divalent and transitional metal ions, interfering with redox activity and protecting against metal-induced oxidative damage [42, 43]. Previous studies have shown that GA may protect the kidneys against various nephrotoxic agents [44, 45]. Although it is considered safe at low to moderate doses, high concentrations of GA have shown potential pro-oxidant or cytotoxic effects in certain in vitro settings [46]. Therefore, dose optimization is critical. In a subchronic rat study, dietary GA at 0.2% of feed (~ 119 mg/kg/day in males) was identified as a No Observed Adverse Effect Level (NOAEL) over 13 weeks [47]. GA oral administration up to 200 mg/kg b.wt for up to 10 weeks in rats showed beneficial biological activities without any adverse effects [48–51]. Moreover, GA holds the Generally Recognized As Safe (GRAS) status by the USFDA, indicating a favorable safety profile with low neurotoxicity and mortality at acute doses across various animal models [52].

Therefore, in this study, we aimed to investigate the renal impact of individual and combined exposure to ATO and ZNPs in rats. Furthermore, we evaluated whether oral supplementation with GA could alleviate oxidative renal dysfunction induced by these toxicants. The study also explored how the different physicochemical forms (micro vs. nano) of the toxicants affect their biological interactions and response to GA intervention.

## Materials and methods

### Tested compounds

Arsenic trioxide (ATO, molecular weight (MW) = 197.84, CAS 1327-53-3, and 99% purity), GA (C_7_H_6_O5. H_2_O, MW = 188.14, CAS Number 5995‑86‑8, and extra‑pure grade, ≥ 99.5% HPLC assay), and ZNPs (MW = 81.39, CAS number: 1314-13-2, an average nano-size of 30 ± 5 nm, and 99% purity) were acquired from Alpha Chemica in Mumbai, India. The analytical quality reagents and chemicals used in this experiment were all obtained from Sigma Company in St. Louis, MO.

GA was dissolved in distilled water, while ZNPs were prepared in distilled water at a concentration of 10 mg/mL, following the method described by Ramadan, et al. [53]. A new suspension was prepared each day, and the suspensions underwent sonication for 15 min. Before administering to the animals, the ZNPs were vortex-mixed, a process previously shown to ensure consistent Zn dispersion [54].

### Experimental animals

Sixty mature male Sprague Dawley rats (average weight 160.33 ± 1.03 and approximately 6 weeks old) were obtained from the breeding facility of the National Research Center (Giza, Egypt). All rats were accommodated in hygienic, well-ventilated steel mesh cages, under a 12-hour light-dark cycle at temperatures between 21 and 24 °C and relative humidity levels of 50 to 60%. Wood shavings were employed to preserve the cages’ dryness. Rats were provided unrestricted access to tap water and commercial standard rat chow (20.3% proteins, 5% fat, 65% carbohydrates, 3.7% salt mixture, 5% fiber, and 1% vitamin mixture) obtained from El Gomhouria Company (Cairo, Egypt). After a two-week acclimatization period, the rats were ready to undergo testing. In compliance with the National Institutes of Health Guide for the Care and Use of Laboratory Animals in Scientific Research, the experimental protocol was approved by Cairo University’s research committee on animal ethics (reference number VET CU 2009 2022461). Moreover, all techniques were recorded in accordance with ARRIVE principles [55]. Rats were weighed and randomly sorted into six groups (*n* = 10 per group). The control group was administered 1 mL of distilled water daily for each rat. The GA group received GA at a dose of 20 mg/kg b.wt, administered orally once daily. Gallic acid was dissolved in distilled water as the vehicle. This dosage and administration route were adopted based on the protocol described in Yigitturk, et al. [56]. The ZNPs group received an oral dosage of 100 mg ZNPs per kg of b. wt [57]. ATO group: administered orally at a dosage of 8 mg/kg b.wt of ATO dissolved in distilled water [58]. The ZNPs + ATO group received co-administration of ZNPs and ATO via identical previous routes and dosages, with a 5-minute delay between the treatments. GA + ZNPs + ATO group: ZNPs and ATO were co-administered sequentially with a 5-minute interval, followed by GA after 1 h, utilizing identical techniques and dosages. The rats were daily administered the tested compounds orally for sixty days via orogastric gavage using a feeding needle (16 gauge). Every week, the dose quantities were adjusted based on the rats’ reported average weights.

### Sampling

Following a 24-hour fast, the rats in each group were anesthetized by intraperitoneal injection of pentobarbital sodium (100 mg/kg body weight). This dosage meets the minimum threshold outlined in the AVMA Guidelines for the Euthanasia of Animals [59], which designates IP pentobarbital between 60 and 100 mg/kg as an acceptable method for rodent euthanasia/anesthesia, and ≥ 100 mg/kg as sufficient for anesthetic overdose. Anesthesia depth was confirmed by loss of pedal and corneal reflexes before terminal procedures. Blood was collected from the retro-orbital venous plexus into a standard tube and allowed to stand at ambient temperature for 20 min. The tubes underwent centrifugation for 10 min at 3000 rpm, and the serum was subsequently stored at -20 °C for future biochemical analysis. The rats were decapitated, and the kidneys were collected and rinsed with saline solution. The kidney specimens were classified into three groups. The first group was preserved in a 10% buffered neutral formalin solution for both histopathological and immunohistochemical analysis. The second group of kidney samples was homogenized using a Potter-Elvehjem rotor-stator homogenizer (Thomas Scientific, Swedesboro, NJ, USA) in ice-cold phosphate-buffered saline. The homogenate was centrifuged for 10 min at 4 °C and 3000 rpm. The supernatants were subsequently assessed for the biochemical tests specified below. The third group of kidney samples was stored at 4 °C until the analysis and evaluation of As and Zn content were conducted.

### Evaluation of serum renal function indicators and electrolytes

Serum analytes were quantified using Spinreact kits (Spinreact S.A., Sant Esteve De Bas, Spain) per manufacturer protocols. Creatinine (MIBSIS13 I) was measured via kinetic Jaffé reaction at 37 °C with absorbance at 492 nm; intra- and inter-assay CVs were approximately 2.8% and 3.9%, respectively. Urea (BSIS35- I) employed the o-phthalaldehyde colorimetric method, with readings at 510 nm following kinetic (A₂–A₁) or end-point (15 min) protocols. Uric acid (MDBSIS45 I) was assessed enzymatically (uricase–peroxidase chromogen reaction) at approximately 520 nm. Total protein (BSIS30 I) was measured using the Biuret reaction (approximately 540 nm), while albumin (BSIS02 I) was measured with bromocresol green dye (approximately 628 nm); globulin levels were derived by subtraction. Calcium (Ca) (MDBSIS09 I), potassium (K) (BSIS53 I), and sodium (Na) (BSIS54 I) were determined via standard colorimetric assays (complexone, tetraphenylborate, and thiocyanate–mercuric methods, respectively) with manufacturer-specified wavelengths.

### Assessment of renal lipid peroxidation and oxidative stress

Kidney homogenates were prepared and analyzed for oxidative stress markers using commercial kits. Malondialdehyde (MDA) levels were quantified using the Biodiagnostic TBARS kit (Cat. MD 25 29, Dokki, Giza, Egypt), incubating samples with thiobarbituric acid at 95 °C for 30 min, and measuring absorbance at 534 nm. Superoxide dismutase (SOD) activity was determined using the Biodiagnostic colorimetric kit (Cat. SD 25 21), with inhibition of dye reduction measured at 560 nm. Glutathione peroxidase (GPx) activity was assessed with the EnzyChrom™ GPx Assay Kit (EGPX‑100; BioAssay Systems, CA, USA), which coupled GPx with glutathione reductase and monitored NADPH consumption at 340 nm over a 20‑min reaction using ~ 10 µL homogenate; the kit’s detection range is 40–800 U/L with a lower limit of ~ 12 U/L. All assays were performed in triplicate, with calibration standards and quality controls to monitor intra‑ and inter‑assay variability.

### Examination of renal concentrations of Zn and As

The kidney samples were microwave-digested using 8 mL of nitric acid, 1 mL of hydrogen peroxide (H_2_O_2_), and 30% of the sample volume. The amounts of Zn and As were measured using an inductively coupled plasma-optical emission spectrophotometer. A Synchronous Vertical Dual View was a feature of the Agilent model 5100, which was manufactured in Santa Clara, CA. For every dataset, an intensity calibration curve was made by combining a blank with a minimum of three Merck Company standards. The precision and reliability of the metal analyses were ensured by utilizing external Merck reference standards. Furthermore, to confirm the accuracy of the equipment, samples of trace element quality control were compared to standards set by NIST. Comparing certified values with analysis of the reference sample yielded recovery rates of 98% for Zn and 97% for As.

### Histopathological evaluations

Kidneys of rats were immediately fixed in neutral buffered formalin (10%) for at least 24 h followed by processing in grades of alcohols, xylene, and melted paraffin wax. The processed tissue samples were inserted in paraffin blocks and sectioned into 5 μm slices for staining with hematoxylin and eosin (H&E) for light microscopy [60]. A Leica DM4B light digital microscope (Leica, Germany) coupled with a Leica DMC4500 digital camera was utilized to analyze slides and capture photographs.

### Immunohistochemical analysis

Five µm slices were affixed on adhesive slides for immunostaining. Subsequently, following the rehydration of tissue sections in distilled water, the sections were incubated with primary antibodies, specifically anti-nuclear factor erythroid 2-related factor 2 (Nrf2) (Proteintech, Germany) and anti-heat shock protein (HSP-90) (Proteintech, Germany), at a dilution of 1:300 at room temperature for one hour in a humid environment. Following extensive washing, tissue pieces were treated with H_2_O_2_ for 10 min. The HRP-labeled secondary detection kit (BioSB, USA) was used to execute the reaction according to the manufacturer’s guidelines. Positive control slides were acquired by avoiding incubation with primary antibodies. The positive expression was measured as the mean area percentage in each group.

### Statistical analysis

The Kolmogorov-Smirnov and Levene’s tests were used to confirm the data’s normal distribution and homogeneity of variances. Once normality was established, a one-way ANOVA was conducted using SPSS version 14 (SPSS, Chicago, IL, USA). Tukey’s post hoc test was applied for group comparisons when normality assumptions were met. For immunohistochemistry and metal residue analysis, the Kruskal-Wallis test was performed, followed by Dunn’s multiple comparisons test. Statistical significance was set at *P* < 0.05.

## Results

### Effects on the serum protein profile

**Fig. 1:**
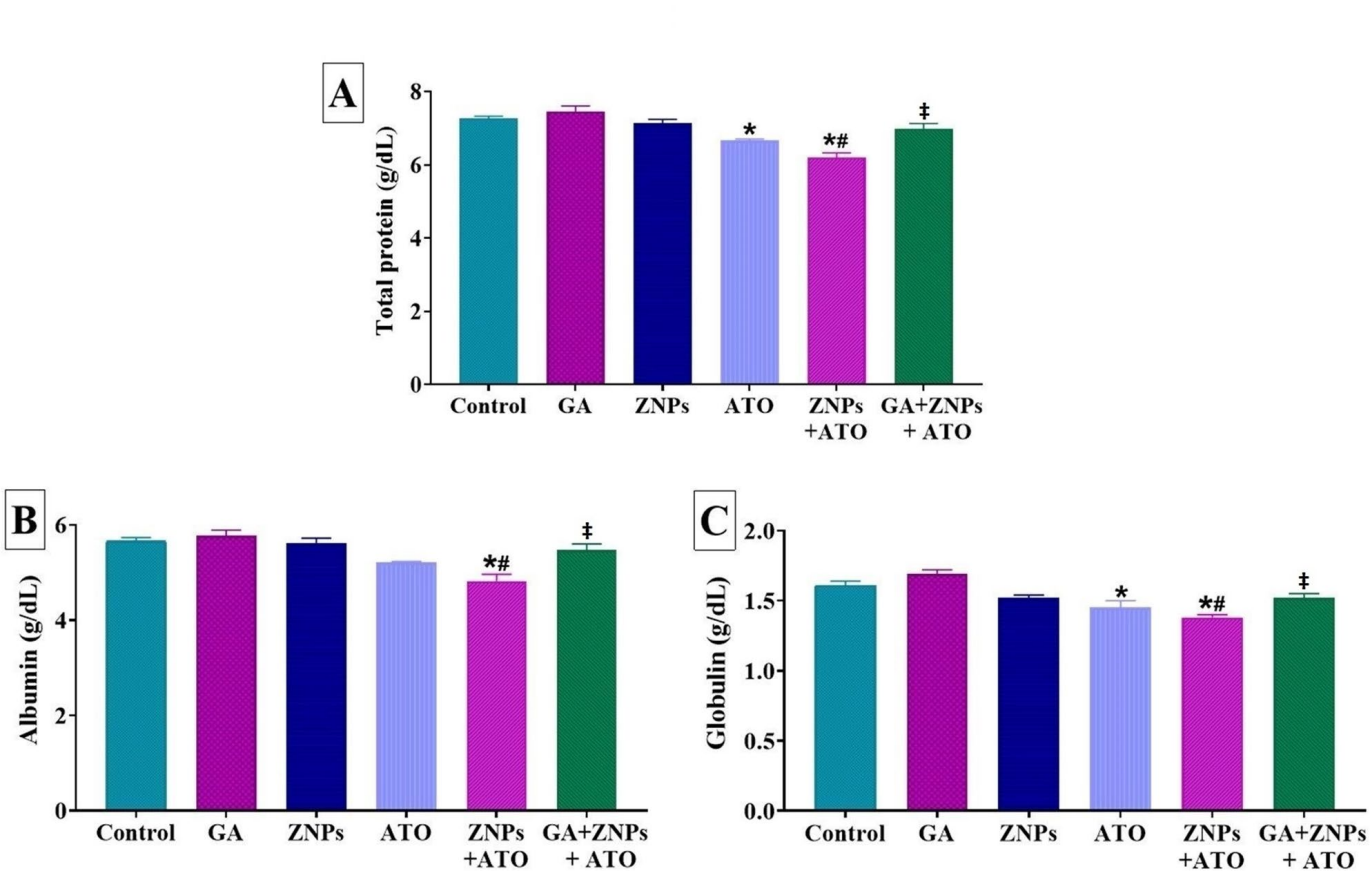
Effects of gallic acid (GA) on serum levels of (**A**) total protein, (**B**) albumin, and (**C**) globulin of rats exposed to zinc oxide nanoparticles (ZNPs) and/or arsenic trioxide (ATO) for 60 days. Data are expressed as the mean ± SE (*n* = 6). * *p* < 0.05 vs. control, # *p* < 0.05 ZNPs + ATO vs. ZNPs, † *p* < 0.05 ZNPs + ATO vs. ATO, and ‡ *p* < 0.05 GA + ZNPs + ATO vs. ZNPs + ATO

Figure 1A-C demonstrates that the protein profile in rats exhibited alterations following a 60-day oral dosing of ZNPs and/or ATO and GA. The groups exposed to ATO demonstrated significantly lower serum levels of total protein (*P* = 0.007) and globulin (*P* = 0.01) in comparison to the control group. Relative to the control group, the ZNPs + ATO co-exposed group exhibited significantly (*P* < 0.001) lowered serum levels of total protein, albumin, and globulin. Rats concurrently exposed to ZNPs and ATO exhibited significantly lower levels of serum total protein (*P* < 0.001), albumin (*P* < 0.001), and globulin (*P* = 0.030) in comparison to those treated with ZNPs alone. In the GA + ZNPs + ATO co-treated group, there was a significant increase in total protein (*P* < 0.001), albumin (*P* = 0.002), and globulin (*P* = 0.030) compared to the ZNPs + ATO co-exposed group. In the group co-treated with GA + ZNPs + ATO, the total protein, albumin, and globulin were restored to a level where no significant changes were noted in comparison to the control group.

### Changes in renal function indices

**Fig. 2:**
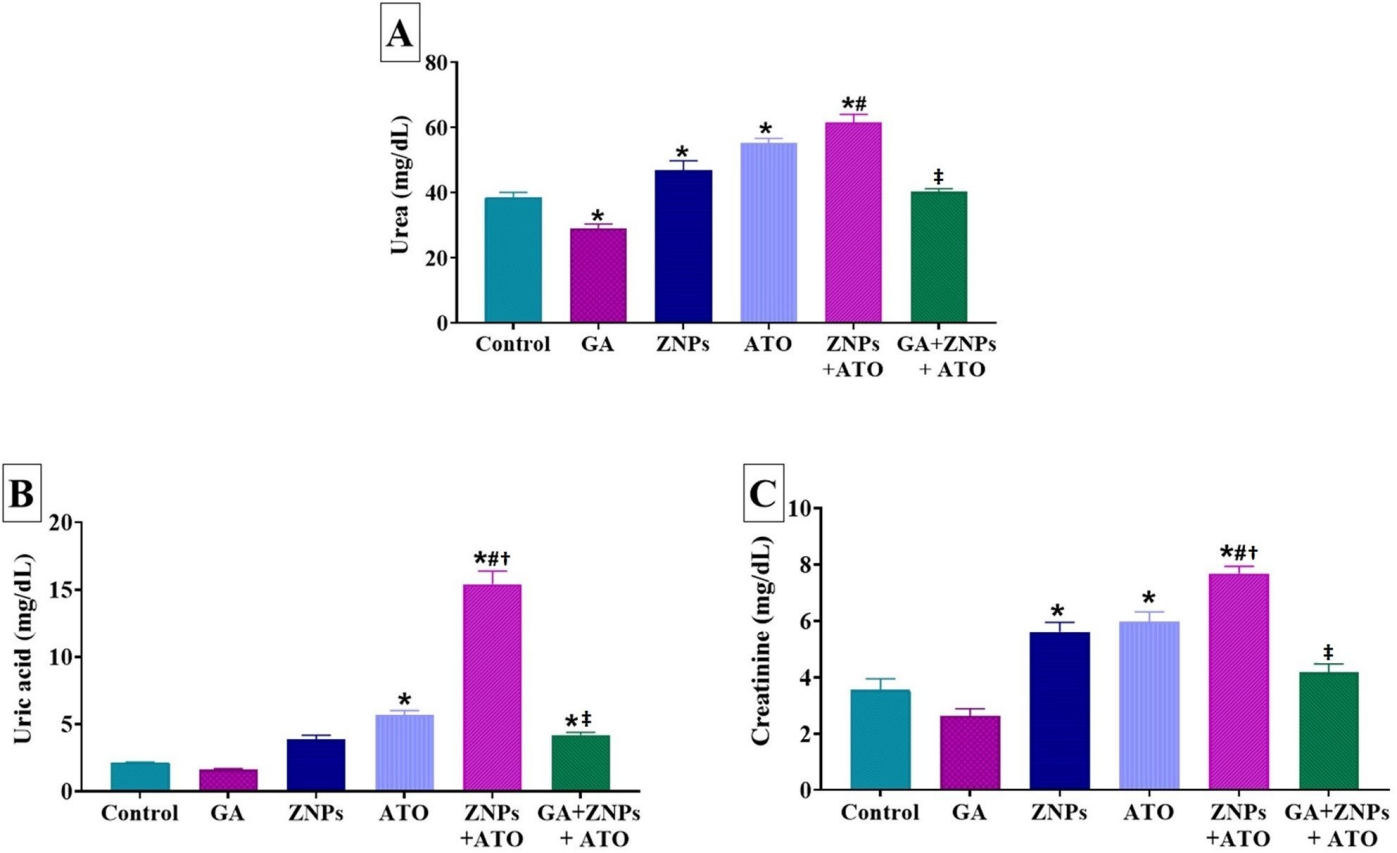
Effects of gallic acid (GA) on serum levels of (**A**) urea, (**B**) uric acid, and (**C**) creatinine of rats exposed to zinc oxide nanoparticles (ZNPs) and/or arsenic trioxide (ATO) for 60 days. Data are expressed as the mean ± SE (*n* = 6). * *p* < 0.05 vs. control, # *p* < 0.05 ZNPs + ATO vs. ZNPs, † *p* < 0.05 ZNPs + ATO vs. ATO, and ‡ *p* < 0.05 GA + ZNPs + ATO vs. ZNPs + ATO

Following a 60-day of sole exposure to ZNPs, there was a significant increase in the serum levels of urea (*P* = 0.033) and creatinine (*P* < 0.001) compared to the control group (Fig. 2A and C). Besides, both the ATO-exposed group and ZNPs and ATO co-exposed one displayed a significant (*P* < 0.001) increase in serum levels of urea, uric acid, and creatinine compared to the control group (Fig. 2A and B, and C). Moreover, the group co-exposed to ZNPs and ATO exhibited a significant (*P* < 0.001) increase in the serum levels of urea (*P* < 0.001), uric acid (*P* < 0.001), and creatinine (*P* = 0.001), relative to the groups individually exposed to ZNPs. Furthermore, the ZNPs and ATO co-exposed group exhibited a significant increase in the serum levels of uric acid (*P* < 0.001) and creatinine (*P* = 0.007), relative to the groups independently exposed to ATO. On the contrary, the serum levels of uric acid, urea, and creatinine were significantly (*P* < 0.001) reduced in the GA + ZNPs + ATO co-treated group compared to the ZNPs + ATO co-exposed group, with no significant changes observed in urea and creatinine serum levels compared to the control group.

### Effects on electrolyte balance

**Fig. 3:**
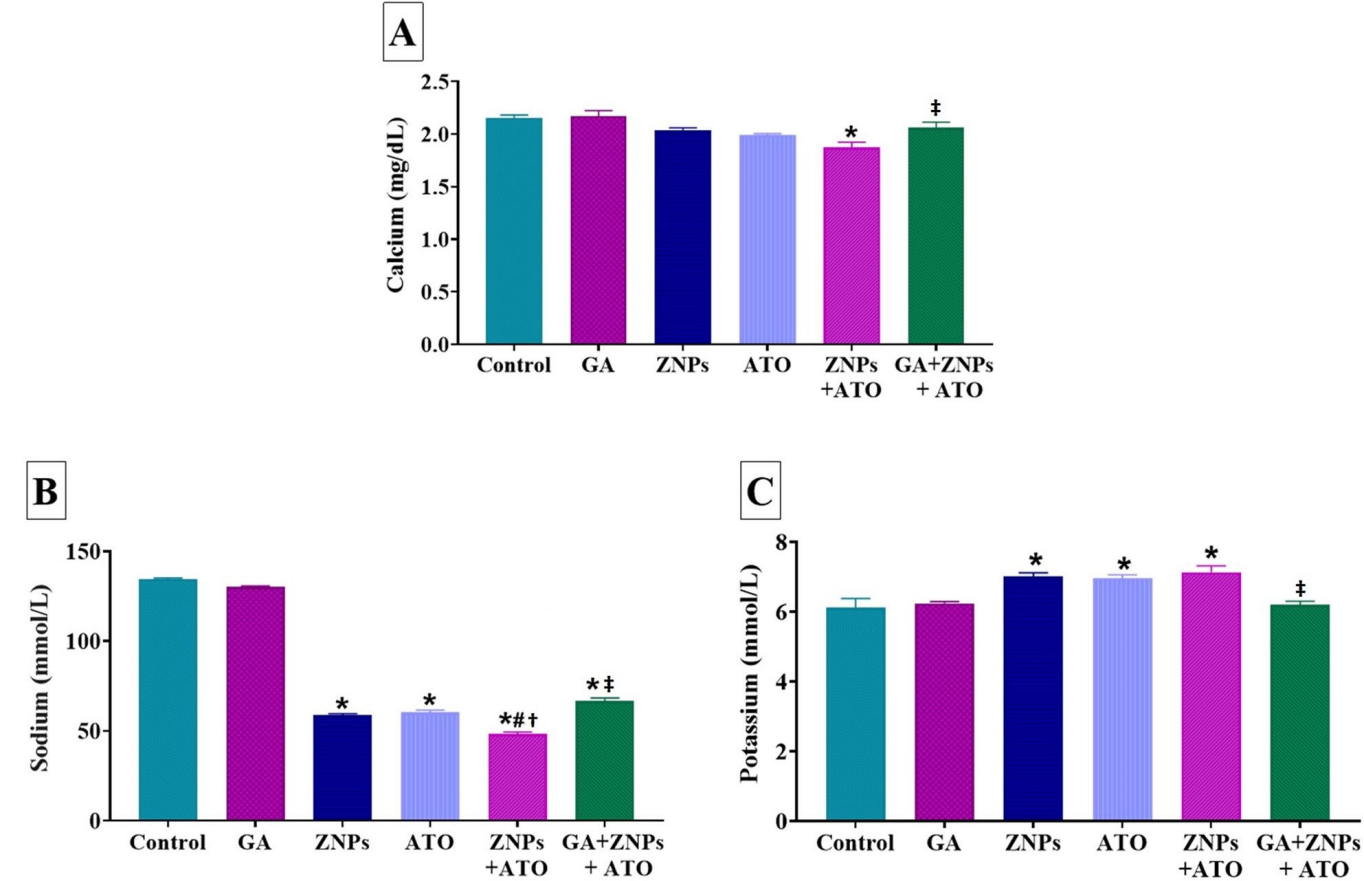
Effects of gallic acid (GA) on serum levels of (**A**) calcium, (**B**) sodium, and (**C**) potassium of rats exposed to zinc oxide nanoparticles (ZNPs) and/or arsenic trioxide (ATO) for 60 days. Data are expressed as the mean ± SE (*n* = 6). * *p* < 0.05 vs. control, # *p* < 0.05 ZNPs + ATO vs. ZNPs, † *p* < 0.05 ZNPs + ATO vs. ATO, and ‡ *p* < 0.05 GA + ZNPs + ATO vs. ZNPs + ATO

Concerning alterations in serum Ca levels, as shown in Fig. 3A, the group exposed to ZNPs + ATO revealed a significant (*P* < 0.001) reduction when compared to the control group. Nevertheless, the GA + ZNPs + ATO co-treated group exhibited a significant (*P* = 0.023) recovery of serum Ca levels compared to the ZNPs + ATO co-exposed group.

Regarding the variations in serum Na levels, as illustrated in Fig. 3B, a significant decrease (*P* < 0.001) in the serum Na level was observed in the rats that were either individually or concurrently exposed to ZNPs and ATO compared to the control group. The group exposed to both ZNPs + ATO showed a significant (*P* < 0.001) decrease in serum Na levels compared to those exposed to either ZNPs or ATO alone. Yet, the group given GA + ZNPs + ATO showed a significant (*P* < 0.001) rise in serum Na levels compared to the group exposed to ZNPs + ATO.

As shown in Fig. 3C, there was a notable increase in the serum K level in ZNPs (*P* = 0.003), ATO (*P* = 0.005), and ZNPs + ATO (*P* = 0.001) groups compared to the control group. However, treatment with GA significantly (*P* = 0.001) ameliorated the observed increased K level in the serum of the co-exposed group, as indicated in Fig. 3C.

### Changes in renal oxidative status

**Fig. 4:**
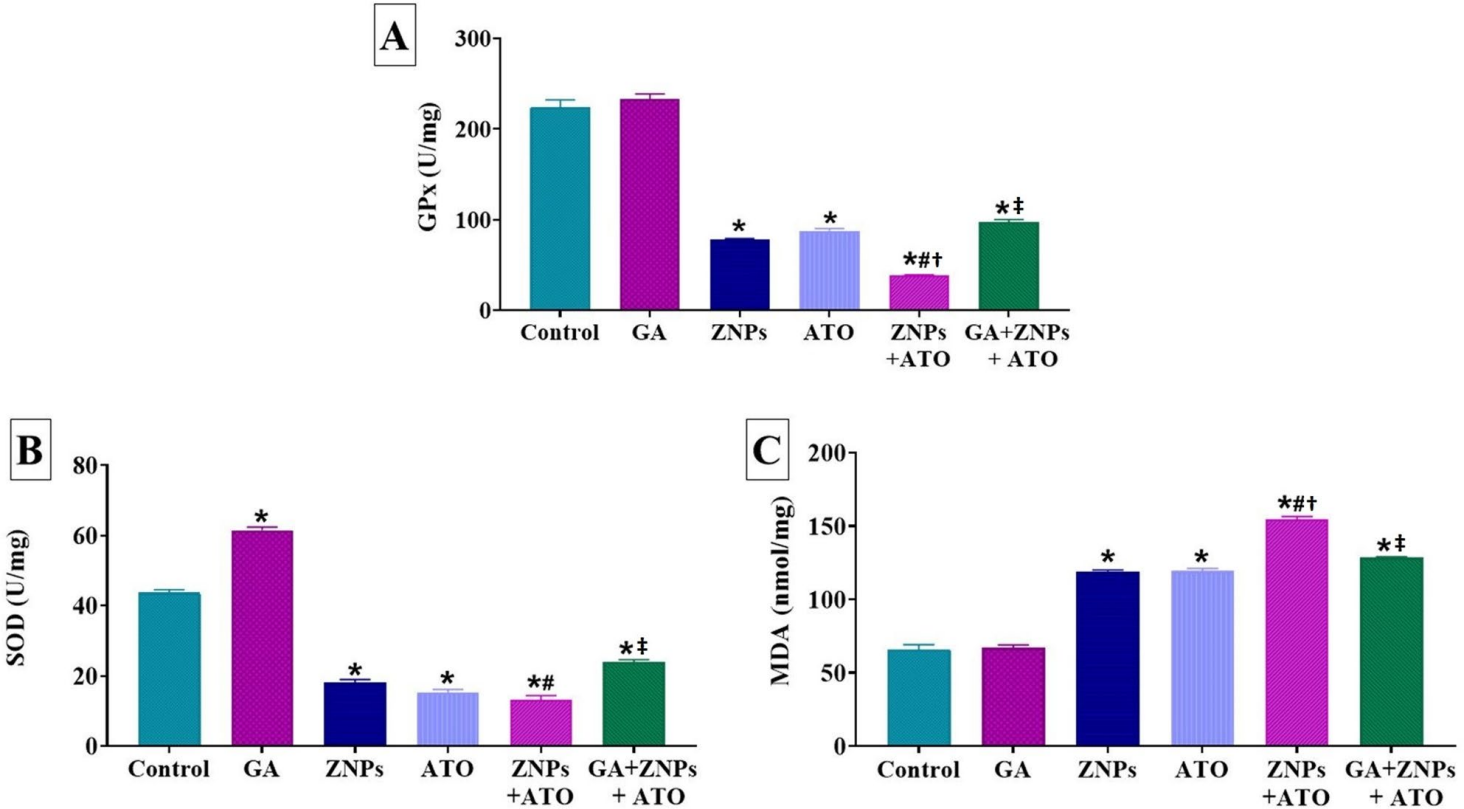
Effects of gallic acid (GA) on antioxidant enzymes and lipid peroxidation indicators including (**A**) glutathione peroxidase (GPx), (**B**) Superoxide dismutase (SOD), and (**C**) malondialdehyde (MDA) indicators in kidney homogenate of rats exposed to zinc oxide nanoparticles (ZNPs) and/or arsenic trioxide (ATO) for 60 days. Data are expressed as the mean ± SE (*n* = 6). * *p* < 0.05 vs. control, # *p* < 0.05 ZNPs + ATO vs. ZNPs, † *p* < 0.05 ZNPs + ATO vs. ATO, and ‡ *p* < 0.05 GA + ZNPs + ATO vs. ZNPs + ATO

Figure 4B demonstrated that daily oral dosing of GA for 60 days significantly (*P* < 0.001) increased SOD activities in kidney tissues relative to the control group. ZNPs and/or ATO-exposed rats had a significant (*P* < 0.001) decrease in SOD and GPx antioxidant enzyme levels, coupled with an increase in MDA relative to the control group (Fig. 4A, B, and C). Significant alterations were observed in the levels of SOD, GPx, and MDA between rats treated solely with ZNPs and those co-exposed to both ZNPs + ATO. Furthermore, the group exposed to both ZNPs and ATO demonstrated a notable (*P* < 0.001) decrease in renal GPx activity, alongside an increase in renal MDA content when compared to the group exposed solely to ATO. In the GA + ZNPs + ATO co-treated group, SOD and GPx activities were significantly (*P* < 0.001) higher compared to the ZNPs and ATO co-exposed group, while MDA levels were significantly (*P* < 0.001) lowered in the latter group.

### Effects of treatments on As and Zn renal accumulation

**Fig. 5:**
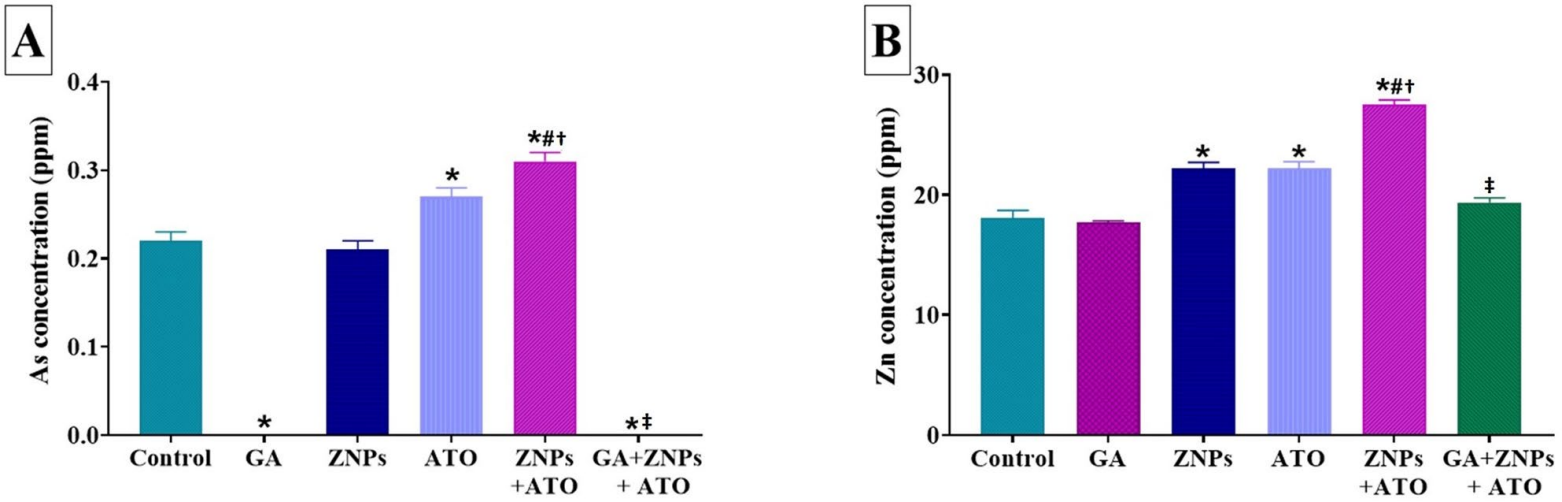
Effects of gallic acid (GA) on arsenic (As) and zinc (Zn) residues in the kidney homogenate of rats exposed to zinc oxide nanoparticles (ZNPs) and/or arsenic trioxide (ATO) for 60 days. Data are expressed as the mean ± SE (*n* = 6). * *p* < 0.05 vs. control, # *p* < 0.05 ZNPs + ATO vs. ZNPs, † *p* < 0.05 ZNPs + ATO vs. ATO, and ‡ *p* < 0.05 GA + ZNPs + ATO vs. ZNPs + ATO

The GA-treated group displayed a significant (*P* < 0.001) reduction in renal As content relative to the control group (Fig. 5A). On the contrary, significantly higher concentrations of renal As were recorded in the ATO group (*P* = 0.003) and ZNPs + ATO (*P* < 0.001) co-exposed group (Fig. 5A). A significant accumulation of renal As was observed in the group co-exposed to ZNPs and ATO, in contrast to the groups exposed to ZNPs (*P* < 0.001) or ATO (*P* = 0.026) alone. In the GA + ZNPs + ATO co-treated group, a significant (*P* < 0.001) reduction of renal accumulation of As was recorded compared to the ZNPs and ATO co-exposed group.

Figure 5B revealed that a significant (*P* < 0.001) increase in renal Zn content was recorded in ZNPs, ATO, and ZNPS + ATO exposed groups relative to the control group. Additionally, a significant (*P* < 0.001) accumulation of renal Zn was detected in the ZNPs and ATO co-exposed group compared to the groups exposed to ZNPs or ATO alone. In contrast to the ZNPs + ATO co-exposed group, oral administration of GA significantly (*P* < 0.001) reduced renal accumulation of Zn. Of notes, the reduction in Zn accumulation in the GA + ZNPs + ATO co-treated group was significant, showing no notable differences when compared to the control group.

### Histopathological findings

**Fig. 6:**
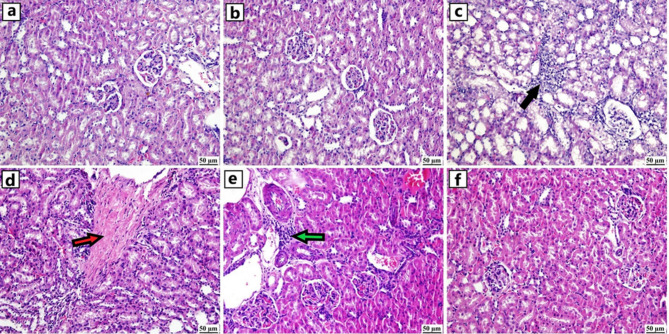
Photomicrographs of kidneys (H&E) (**a**) Control and (**b**) Gallic acid (GA) groups showing normal structure of kidney, (**c**) Zinc oxide nanoparticles (ZNPs) group showing focal aggregation of mononuclear inflammatory cells (black arrow), (**d**) Arsenic trioxide (ATO) group showing fibroplasia (red arrow) with mononuclear inflammatory cells infiltration, (**e**) ZNPs + ATO group showing perivascular mononuclear inflammatory cells infiltration (green arrow) and (**f**) GA + ZNPs + ATO group showing apparently normal renal tissue

The examined kidney sections from both control and GA groups revealed the normal structure of renal parenchyma, including renal tubules and glomeruli. On the contrary, ZNPs group exhibited interstitial nephritis manifested by the presence of focal aggregations of mononuclear inflammatory cells. The renal tubular epithelium suffered from degenerative changes. Likewise, the ATO group showed nephritis with perivascular fibroplasia in some instances. ZNPs + ATO group showed exaggerated damage represented by interstitial damage, renal tubular necrosis, and vascular wall thickening. The GA + ZNPs + ATO group showed obvious amelioration of renal toxicity. Histological examination revealed that while some renal sections exhibited mild degeneration of the tubular epithelium, other sections appeared histologically normal (Fig. 6).

### Immunohistochemical expression of Nrf2 and HSP90 in renal tissue

#### Nrf2 expression

**Fig. 7:**
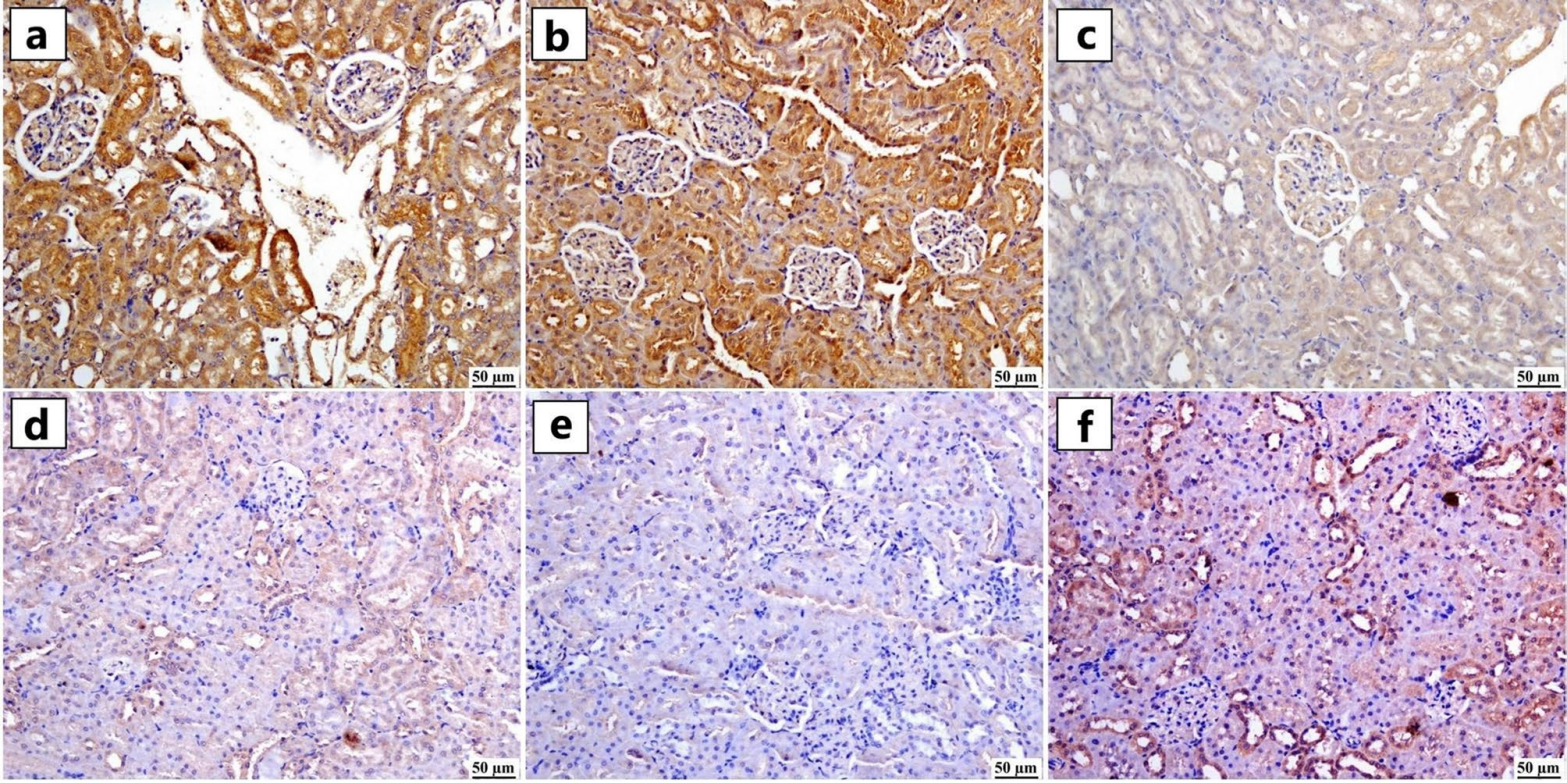
Photomicrographs of kidneys (Immune staining) showing Nuclear factor erythroid 2-related factor 2 (Nrf2) expression. (**a**) control and (**b**) Gallic acid (GA) groups showing intense Nrf2 expression, (**c**) Zinc oxide nanoparticles (ZNPs) and (**d**) Arsenic trioxide (ATO) groups showing mild Nrf2 expression, (**e**) ZNPs + ATO groups showing limited to negative immune staining, and (**f**) GA + ZNPs + ATO group showing a moderate level of Nrf2

Immunohistochemical staining for Nrf2 (Fig. 7a-f) revealed strong nuclear and cytoplasmic expression in the renal tubular epithelium of the control and GA-treated groups (Figs. 7a and 7b), as evidenced by intense brown immunoreactivity. In contrast, ZNPs and ATO groups (Figs. 7c and 7d) exhibited markedly diminished Nrf2 expression. The ZNPs + ATO co-exposed group showed the most pronounced reduction (Fig. 7e). However, the GA + ZNPs + ATO-treated group (Fig. 7f) demonstrated partial restoration of Nrf2 expression, with some renal tubules displaying moderate immunoreactivity and others appearing near-normal. Quantitative analysis (Fig. 9A) showed that the Nrf2-positive area was significantly reduced in the ZNPs, ATO, and ZNPs + ATO groups compared to the control and GA groups (*P* < 0.05). A significant (*P* < 0.05) reduction of Nrf2 expression was detected in the ZNPs and ATO co-exposed group compared to the groups exposed to ZNPs or ATO alone. Yet, the GA + ZNPs + ATO-treated group showed a significant increase in Nrf2 expression compared to the ZNPs + ATO co-exposed group, though it did not fully return to control levels.

#### HSP90 expression

**Fig. 8:**
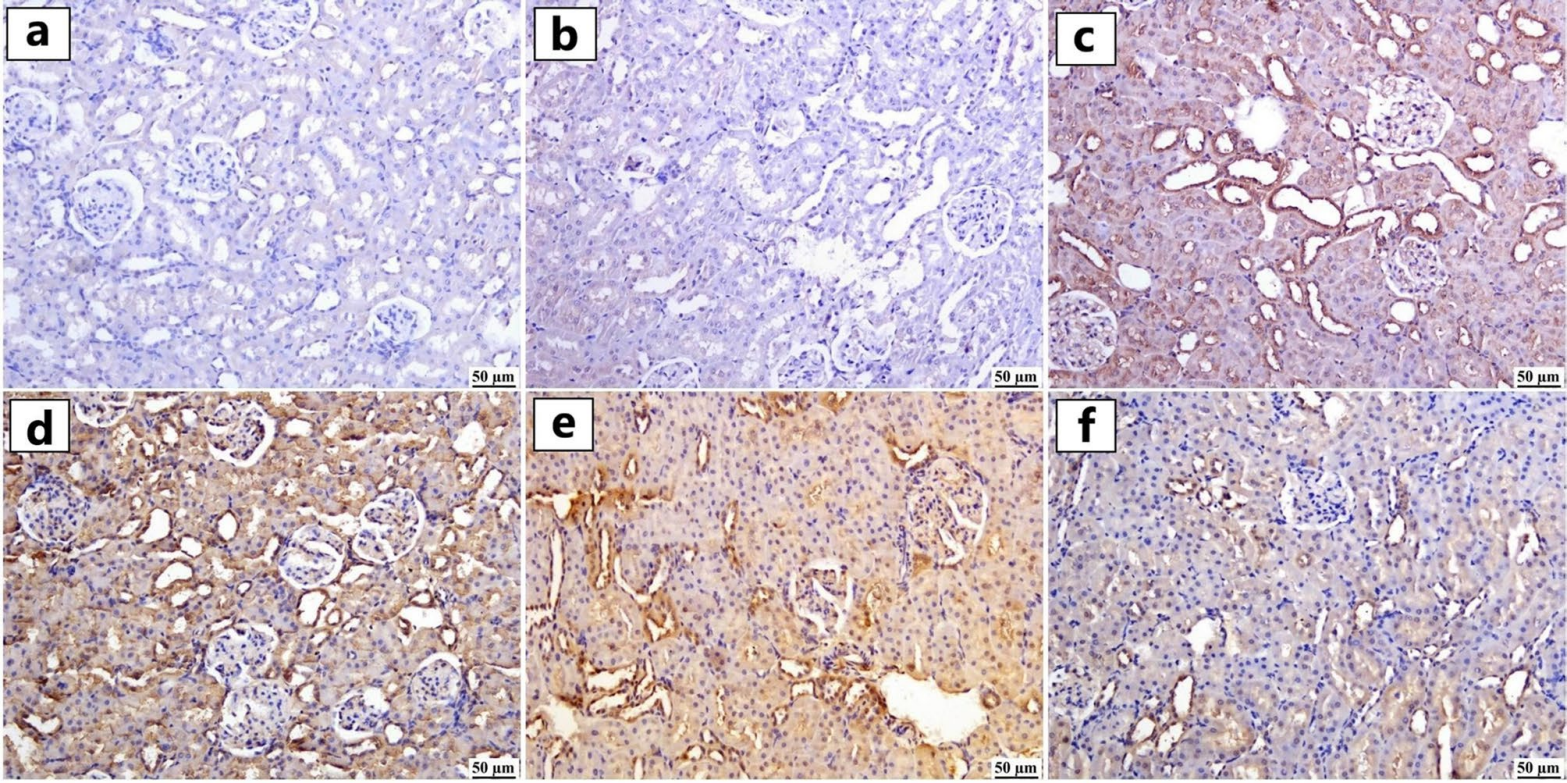
Photomicrographs of kidneys (Immune staining) showing Hsp90 heat shock protein 90 (HSP90) expression. (**a**) control and (**b**) Gallic acid (GA) groups showed limited HSP-90 expressions, (**c**) Zinc oxide nanoparticles (ZNPs), (**d**) Arsenic trioxide (ATO), and (**e**) ZNPs + ATO groups showed increased HSP-90 expression, and (**f**) GA + ZNPs + ATO group showed a mild level of HSP90

**Fig. 9:**
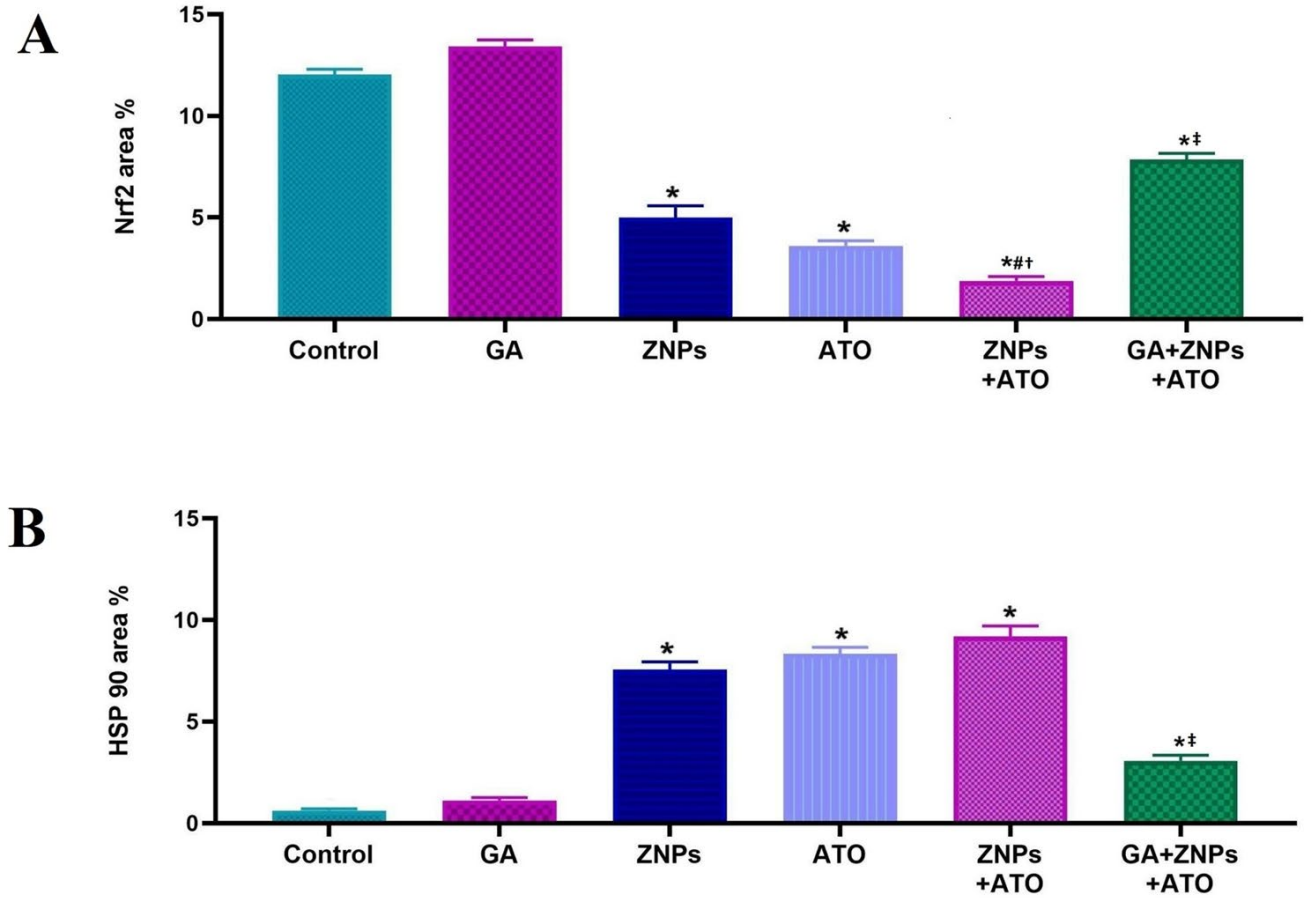
Renal immunoexpression of (**A**) erythroid 2-related factor 2 (Nrf2) and (**B**) heat shock protein 90 (HSP-90) in rats exposed to zinc oxide nanoparticles (ZNPs) and/or arsenic trioxide (ATO) for 60 days. Data are presented as mean ± SE. * *p* < 0.05 vs. control, # *p* < 0.05 ZNPs + ATO vs. ZNPs, † *p* < 0.05 ZNPs + ATO vs. ATO, and ‡ *p* < 0.05 GA + ZNPs + ATO vs. ZNPs + ATO

As shown in Fig. 8a–f, HSP90 expression was minimal in the control and GA groups (Figs. 8a and 8b). Yet, ZNPs, ATO, and especially the ZNPs + ATO co-exposed group (Fig. 8c–e) exhibited marked upregulation of HSP90 expression, predominantly localized in renal tubules. Interestingly, in the GA + ZNPs + ATO group (Fig. 8f), HSP90 expression was substantially reduced compared to the ZNPs + ATO co-exposed group. Quantitative assessment (Fig. 9B) revealed a significant increase in HSP90-positive area in the ZNPs, ATO, and ZNPs + ATO groups (*P* < 0.05), while the GA + ZNPs + ATO-treated group showed a significant reduction compared to the ZNPs + ATO co-exposed group, though still increased relative to control.

## Discussion

Numerous prior studies have accepted impaired serum creatinine, urea, and uric acid as dependable nephrotoxicity markers [38, 61, 62]. After 60 days of oral dosing with ATO and/or ZNPs, there was a significant increase in creatinine, uric acid, and urea levels, indicating obvious renal damage. The histological observations confirmed these results, revealing significant degenerative changes and chronic interstitial nephritis in the groups treated with ATO and/or ZNPs. Consequently, a notable As and Zn accumulation was observed in the renal tissues of ATO and/or ZNP-exposed rats. Injuries to the kidneys resulting from the accumulation of As and Zn may lead to inflammatory cell activation in the interstitial spaces or glomeruli. The cells successively generate ROS and inflammatory cytokines [63, 64]. Herein, the ATO and/or ZNPs-exposed group demonstrated a considerable increase in the HSP90 immunoexpression in their renal tissues, reflecting the progression of inflammation. HSP90 serves as a chaperone that enhances the functionality of various critical proinflammatory molecules associated with abnormal inflammation [65]. Additionally, HSP90 expression is increased in response to cellular stress, diseases, or an increased demand for protein synthesis to sustain cellular function [66]. Conversely, a significant protective effect was observed in the GA-treated groups, evidenced by reduced serum levels of renal damage markers, accumulated As and Zn, and HSP90 immunoexpression, alongside restoration of renal architecture. Similarly, GA preserved kidney architecture and decreased serum levels of renal damage products associated with diclofenac administration [44] and paraquat exposure [45] in rats. This may primarily pertain to the diminished accumulation of As and Zn in the GA-treated groups, likely attributable to its chelating effect [67]. Deguchi [68] assessed the interaction between Zn and GA using potentiometric titration methods and demonstrated significant complexation between the two, indicating that Zn exhibits a preference for bonding to the GA carbonyl group. Furthermore, the antioxidant properties of GA may play a central role in preserving tubular architecture and inhibiting the leakage of creatinine, uric acid, and urea into the bloodstream. The antioxidant activity is linked to phenolic compounds, which are characterized by their chemical structure and reducing properties. These compounds play a key role in the sequestration of free radicals and the chelation of transition metals [67]. Furthermore, the inhibition of HSP90 induced by GA may have contributed to the reduction of inflammation. Inhibition of HSP90 has been explored as a strategy for developing therapies aimed at addressing renal diseases linked to inflammation [69–71].

Significant hypoproteinemia, hypoalbuminemia, and hypoalbuminemia were observed in rats exposed to ATO and ZNPs. Multiple factors may lead to reduced serum globulin, albumin, and total protein levels in cases of kidney damage induced by ATO and ZNPs. This includes proteinuria and the associated loss of albumin and other proteins from the blood, resulting in diminished serum globulin, albumin, and total protein levels [64, 72]. Furthermore, the in vitro study conducted by Shim, et al. [73] demonstrated a significant binding of ZNP with various cellular proteins, which may impair the kidney’s capacity to synthesize proteins at the normal rate. Furthermore, Vergara-Gerónimo, et al. [74] indicated that As and its metabolites engage with proteins through direct binding to specific cysteine residues, cysteine clusters, and Zn finger motifs. As a result, the interactions between ATO and ZNPs with proteins interfere with the synthesis and functionality of proteins. In this study, ATO and ZNPs-induced kidney damage was linked to inflammatory reactions, as evidenced histologically by interstitial inflammatory cell infiltrations and immune-histochemically by a significant increase in HSP90 immunoexpression in the kidney. The production of various proteins, such as albumin and globulins, may be impacted, resulting in reduced levels [75, 76]. In contrast, GA oral dosing significantly rectified the decrease in serum protein levels induced by ATO and ZNPs, while also enhancing renal HSP90 expression. The findings indicate that GA has the potential to alleviate inflammatory changes in the kidneys. The GA plays a crucial regulatory role in amino acid metabolism and protein synthesis, mainly through its impact on intestinal health and amino acid uptake, which may be responsible for modulating protein levels [77, 78].

Maintaining a well-mineralized equilibrium is crucial for optimal kidney function [79]. Disruptions in concentrations of serum electrolytes, such as K, Na, and Ca, are recognized to impair renal function [80]. The present study indicates that exposure to ATO and/or ZNPs resulted in notable changes in electrolyte balance, including hypocalcemia (significant only with combined exposure), hyponatremia, and hyperkalemia. Multiple epidemiologic studies in both animals and humans have indicated a correlation between ATO poisoning and hypocalcemia [81, 82]. ATO can disrupt calcium homeostasis by influencing the secretion and action of parathyroid hormone, resulting in reduced calcium absorption in the intestines and heightened renal excretion [83]. Furthermore, the identified degenerative changes in the renal tubules of rats exposed to ZNPs and/ ATO or may result in renal tubular dysfunction, which could hinder Na reabsorption and consequently increase Na loss in urine. Furthermore, oxidative stress induced by arsenic can interfere with the functionality of sodium channels and transporters, worsening conditions such as hyponatremia and hyperkalemia [84, 85]. Furthermore, the differences noted in the electrolyte profiles between the ATO + ZNPs co-exposed group and the groups exposed to ATO or ZNPs individually suggest possible additive or synergistic effects of these substances on electrolyte balance. The hypocalcemia was notably significant solely in the group co-exposed to ATO and ZNPs. This response indicates that the exaggerated inflammatory reaction caused by co-exposure to ATO and ZNPs may disrupt calcium metabolism, potentially leading to hypocalcaemia [86]. However, the precise mechanisms contributing to these electrolyte imbalances may entail intricate interactions and pathways that necessitate additional research. In contrast, rats treated with GA + ATO + ZNPs exhibited a notable correction of the altered electrolyte profile. In comparison, GA rectified the disturbances in serum electrolyte levels, such as potassium, calcium, and magnesium, induced by aluminum chloride in Wistar rats [87]. The authors suggested that GA enhanced the functions of Na/K and Ca/Mg ATPases, which are essential for sustaining electrolyte balance. GA has also been proposed to be advantageous in addressing electrolyte level disturbances owing to its critical role in mitochondrial function, antioxidant effects, and cellular energy production [88]. GA positively influences the mitochondrial electron transport chain by enhancing the activities of cytochrome C oxidase and NADH cytochrome C oxidoreductase [89]. Effective ATP production is essential for the optimal operation of transporters, ion channels, and pumps that play a key role in regulating electrolyte balance [90]. By supporting mitochondrial function, GA may indirectly improve the normal operation of these essential cellular processes [91]. Another important aspect is that oxidative stress-induced damage to cells, including those responsible for electrolyte transport, may influence electrolyte imbalances [92]. Therefore, the antioxidant properties of GA may play a role in its protective effects against oxidative stress, which has the potential to worsen electrolyte disturbances [93].

The capacity of ZNPs and ATO to produce excessive ROS was considered the primary factor contributing to tissue injury [9, 18]. The results of the current study indicate that rats exposed to ZNPs and/or ATO exhibited significant depletion of antioxidant enzymes, accompanied by a marked increase in MDA levels. Both ZNPs and ATO exert their toxic effects through multiple mechanisms that compromise cellular antioxidant defenses and induce oxidative stress. ATO disrupts mitochondrial respiration by uncoupling oxidative phosphorylation and inhibiting electron transport, resulting in the overproduction of ROS, such as superoxide and hydrogen peroxide [94]. Additionally, it enhances ROS generation by upregulating NADPH oxidases [95]. Arsenite, the active form of ATO, binds strongly to thiol (-SH) groups in proteins, directly inhibiting key antioxidant enzymes, including SOD, GPx, glutathione reductase, and thioredoxin reductase [96]. Moreover, through conjugation and methylation reactions, arsenic depletes intracellular glutathione (GSH), a cofactor essential for GPx activity, thereby further reducing antioxidant capacity [97]. ZNPs, on the other hand, generate ROS directly (e.g., superoxide, hydroxyl radicals) within renal cells, and these ROS interact with antioxidant enzyme active sites, leading to oxidative modifications that impair the function of enzymes such as SOD and GPx [98]. Furthermore, a notable decrease in the immunoexpression of Nrf2 was observed in the renal tissues of rats exposed to ZNPs and ATO. Nrf2 serves as a vital regulator of the antioxidant response, the preservation of redox balance, the removal of heavy metals, and the control of inflammation [99]. In a comparable study, Ma, et al. [100] found that contamination of laying hens’ diets with As may induce renal oxidative stress by inhibiting the Nrf2/Keap1 pathway. Similarly, Liu, et al. [101] indicated that ZNPs have the potential to induce oxidative stress and mitochondrial injury in renal tubular cells, resulting in renal dysfunction. In contrast, GA markedly diminished ZNPs and ATO-induced oxidative stress. The GA structure features an aromatic ring that includes three hydroxyl groups and one carboxylic acid group. The presence of these phenolic (-OH) groups, along with their planar arrangements, contributes to the free radical scavenging properties of GA [102]. Furthermore, the metal chelating properties of GA, particularly concerning Fe^3+^ ions, are consistently utilized to prevent the Fenton-like reaction and reduce the likelihood of free radical generation, such as hydroxyls (OH−) [103]. Notably, the group treated with GA alone exhibited a significant increase in renal SOD activity. While the increased activity of antioxidant enzymes may represent an adaptive response to oxidative stress, the simultaneous reduction in MDA levels suggests that GA effectively mitigates oxidative damage. These findings indicate that GA not only lowers the pro-oxidant burden but also strengthens the kidney’s antioxidant defense system [45, 104].

In the current study, minimal amounts of As were detected in the renal tissues of both the control and ZNP-only groups. Consistent with these findings, a previous study by Nandi, et al. [105] reported the presence of trace levels of As in various organs, including the liver and kidney, of control animals. This is likely attributable to the widespread distribution of As in the environment, which can lead to background exposure even in the absence of direct experimental dosing [106, 107]. Therefore, the As detected in the control and ZNP-only groups likely reflect baseline levels, rather than experimental dosing. Notably, As was undetectable in the renal tissues of the GA-only treated group, possibly due to GA’s chelating or excretion-enhancing properties, which may facilitate the elimination of trace As from the body [67]. On the contrary, rats exposed individually to ATO or ZNPs exhibited a notable accumulation of renal As and Zn. Prior research on ATO toxicity has demonstrated that the kidney serves as a main organ site for As accumulation [108, 109]. Prior toxicokinetic studies indicated that repeated oral administration of ZNPs may be detrimental, capable of traversing the intestinal barrier, accumulating in various tissues, especially the kidneys, and resulting in adverse effects [64]. Notably, the levels of Zn observed in both the ATO and ZNP groups were comparable, despite the ATO group not being exposed to ZNPs. This similarity suggests that ATO exposure alone may influence systemic Zn distribution. Several mechanisms could underlie this effect, including the modulation of metal homeostasis and physiological compensation. In this context, a study in rats exposed to inorganic As demonstrated that arsenic significantly altered metallothionein-1 expression in the liver and kidney, and affected copper concentrations in renal tissue, indicating a disruption of metal-regulating proteins and homeostatic mechanisms [110]. Furthermore, As metabolites are actively eliminated via efflux transporters and glutathione conjugation pathways, implying that arsenic exposure may also affect the renal handling and tissue distribution of other metals, including Zn [111]. Additionally, rats subjected to ATO and ZNPs demonstrated increased renal levels of As and Zn compared to those exposed to each pollutant individually. The accumulation of heavy metal ions in mammalian organs, assisted by NPs, may increase toxicity in vivo during co-exposure scenarios [26, 112, 113]. Conversely, oral administration of GA markedly reduced the accumulation of Zn and As in the renal tissues of rats. GA has demonstrated the ability to decrease heavy metal burdens in various tissues, suggesting potential excretion-enhancing or chelating properties [67].

Our findings strongly support the notion that co-exposure to ATO and ZNPs exerts a synergistic nephrotoxic effect. This is evidenced by the greater deterioration in renal function markers, electrolyte imbalance, enhanced oxidative stress (MDA increase and SOD and GPx suppression), and intensified histopathological damage in the co-exposed group compared to individual exposures. Mechanistically, the simultaneous induction of ROS by both ATO and ZNPs likely overwhelms the antioxidant defense systems and exacerbates inflammation, as reflected by increased HSP90 expression and diminished Nrf2 signaling [114]. Importantly, GA administration significantly mitigated this synergistic toxicity. GA attenuated the biochemical and histological damage by restoring antioxidant enzyme activities, reducing lipid peroxidation, normalizing electrolyte and renal function markers, and preserving renal architecture. Notably, GA also downregulated HSP90 expression and partially restored Nrf2 levels, indicating reduced cellular stress and enhanced redox balance. These effects may be attributed not only to GA’s potent antioxidant and anti-inflammatory properties but also to its moderate chelating capacity, which likely limited renal accumulation of As and Zn, thereby blunting their interactive toxicity [42, 115]. Thus, GA interrupts the vicious cycle of ROS generation and inflammatory damage triggered by ATO-ZNP co-exposure, highlighting its potential as a protective agent in mixed-metal environmental toxicity scenarios.

It is crucial to acknowledge that, despite the diverse analyzed indicators evaluating renal function in this work, there is a distinct need for further mechanistic investigations. These investigations should seek to elucidate the various pathways potentially associated with the nephron-protective effects of GA, chiefly focusing on the inflammatory cascade. Nevertheless, in spite of this constraint, our study affords substantial data regarding the GA defensive effects against nephrotoxicity generated by ATO and ZNPs, emphasizing the need for additional investigation in this area.

## Conclusions

The current study demonstrated the adverse impacts of ATO and ZNPs exposure on renal function, as evidenced by alterations in biochemical, histological, and immunohistochemical endpoints, particularly when both agents were present concurrently. GA administration provided effective protection against renal damage induced by combined ATO and ZNPs exposure, likely due to its antioxidant properties and metal-chelating activity. These findings suggest that GA holds promise as a protective dietary supplement for individuals at risk of combined heavy metal and NPs exposure, particularly those in industrial settings. However, we recommend conducting further clinical and translational studies to evaluate the safety, efficacy, and dosing of GA in human populations. Additionally, future investigations are warranted to elucidate the alternative mechanisms underlying GA’s protective actions, including its potential roles in modulating intracellular signaling pathways and enhancing mitochondrial function.

## Data Availability

Data is provided within the manuscript or supplementary information files.
